# Ecological Interactions Shape the Dynamics of Seed Predation in *Acrocomia aculeata* (Arecaceae)

**DOI:** 10.1371/journal.pone.0098026

**Published:** 2014-05-29

**Authors:** Anielle C. F. Pereira, Francine S. A. Fonseca, Gleicielle R. Mota, Ane K. C. Fernandes, Marcílio Fagundes, Ronaldo Reis-Júnior, Maurício L. Faria

**Affiliations:** 1 Department of General Biology, State University of Montes Claros, Montes Claros, Minas Gerais, Brazil; 2 Chemical Institute, State University of Campinas, Campinas, São Paulo, Brazil; 3 Institute of Agricultural Sciences, Federal University of Minas Gerais, Montes Claros, Minas Gerais, Brazil; University of Tartu, Estonia

## Abstract

**Background:**

The complex network of direct and indirect relationships determines not only the species abundances but also the community characteristics such as diversity and stability. In this context, seed predation is a direct interaction that affects the reproductive success of the plant. For *Acrocomia aculeata*, the seed predation by *Pachymerus cardo* and *Speciomerus revoili* in post-dispersal may destroy more than 70% of the propagules and is influenced by the herbivory of the fruits during pre-dispersal. Fruits of plants with a higher level of herbivory during pre-dispersal are less attacked by predators in post-dispersal. We proposed a hypothesis that describes this interaction as an indirect defense mediated by fungi in a multitrophic interaction. As explanations, we proposed the predictions: i) injuries caused by herbivores in the fruits of *A. aculeata* favor fungal colonization and ii) the colonization of *A. acuelata* fruit by decomposing fungi reduces the selection of the egg-laying site by predator.

**Methodology/Principal Findings:**

For prediction (i), differences in the fungal colonization in fruits with an intact or damaged epicarp were evaluated in fruits exposed in the field. For prediction (ii), we performed fruit observations in the field to determine the number of eggs of *P. cardo* and/or *S. revoili* per fruit and the amount of fungal colonization in the fruits. In another experiment, in the laboratory, we use *P. cardo* females in a triple-choice protocol. Each insect to choose one of the three options: healthy fruits, fruits with fungus, or an empty pot. The proposed hypothesis was corroborated. Fruits with injuries in the epicarp had a higher fungal colonization, and fruits colonized by fungi were less attractive for egg-laying by seed predators.

**Conclusion/Significance:**

This study emphasizes the importance of exploring the networks of interactions between multitrophic systems to understand the dynamics and maintenance of natural populations.

## Introduction

Terrestrial ecosystems are characterized by high species richness and a wide range of interactions among these species. These interactions play a central role in the evolution and maintenance of species, ecological succession, and energy fluxes [1]–[3]. Historically, ecological theory has been dominated by studies of the direct interactions between one or two trophic levels, such as plant-herbivore and predator-prey interactions [4]. The main categories of direct interactions involve decomposers, predators, herbivores, parasites, parasitoids, mutualists, and competitors [2], [5]–[7]. The categories of interactions are usually defined as positive, negative, or neutral based on their direct effect on the growth or mortality of each species involved [6].

However, ecological interactions between two species can often be indirectly mediated by a third species [3], [8]–[11]. This type of interaction is described in the literature mainly regarding plant defenses against herbivores (performed by ants) [12]–[16], the attraction of parasitoids by volatile substances produced by plants under attack by herbivores [17], [18], and interactions in which the presence of endophytic fungi, pathogens, or mycorrhizae defends the plants by producing secondary metabolites that have different effects on the performance of herbivores [19]–[23]. These interactions are spatially and temporally dynamic and can be difficult to distinguish [24]. The complex network of direct and indirect interactions determines not only the abundance of a single species [20] but also the characteristics of the community, such as diversity and stability [24]–[28].

Seed predation is an direct interaction that affects reproductive success [29] and competitive ability [30], causing a considerable reduction in the adaptive value of the plant [31], [32]. In these systems, predators may be acting during two distinct moments: pre-dispersal or post-dispersal of the propagule [32]. Predation during pre-dispersal occurs before the seed is released by the mother plant and can occur in ripe and unripe fruits. By contrast, post-dispersal predation occurs when the fruits are already on the soil, and the predators include a vast array of animals with more generalist habits. The great majority of the seeds die due to the action of predators and/or pathogens before germination or even at the seedling stage [33].

There are several records of predation on palm fruits by invertebrates [34]–[38], and regarding the palm *Acrocomia aculeata* (Jacq.) Lodd. ex. Martius (Arecaceae), insect attacks on its fruits have already been reported during pre- and post-dispersal [37], [39]. The attack by insects on fruits during pre-dispersal is restricted to the epicarp and mesocarp of the propagule and is performed mainly by larvae of Coleoptera and Lepidoptera (Author’s personal observation). Therefore, in this study, were considered as herbivory. In contrast, insects that attack fruits in post-dispersal, specifically beetles from the subfamily Bruchinae, destroy the embryo while feeding from on the seed [38], [40], [41]. For this reason, were here considered as predators. Predation in post-dispersal has a large impact on the fitness of the plant because it can destroy most of the propagules [32], [42], [43]. This predation is currently economically relevant as fruits of this palm are considered a promising source for biofuel production.

Previous field observations from our research group have revealed that the attack of Bruchinae beetles on *A. aculeata* seeds is influenced by herbivory rates on the fruits during pre-dispersal. Plants with higher rates of attack by herbivores on fruits that are still attached to the cluster are less likely to have their seeds attacked by predators during post-dispersal. Whereas herbivory of fruits serves as an entrance for colonization by fungi, we propose a hypothesis of indirect defense mediated by the presence of decomposing fungi in a multitrophic interaction network composed by the plant, herbivores, decomposers, and seed predators. As potential explanations, we proposed that: i) injuries in the epicarp, caused by herbivores during pre-dispersal, favor colonization by decomposing fungi and ii) the colonization of the fruit of *A. aculeata* by decomposing fungi reduces its selection by the seed predator as egg-laying site.

## Materials and Methods

### Study System

*A. aculeata* (Arecaceae), commonly known as *macaúba* or *bocaiúva*, is a palm native from savannas of Tropical America [44]. In Brazil, this species is the palm with the third highest distribution range, occurring in natural populations across almost the entire territory [45]. This species occurs naturally in large populations, in both disturbed and undisturbed areas, and is well adapted to different environments [46]. *A. aculeata* is an evergreen, arborescent species that reach up to 16 m in height [47], [48]. Flowering occurs between August and December, and the ripening and fall of the fruits occurs, mostly, between October and January, with possible variations between years. Each cluster produces an average of 60 fruits and can reach up to 270 fruits [49]. The fruit is a globose drupe, composed of a chartaceous epicarp (peel); a thin, mucilaginous, and fibrous mesocarp (pulp); and a hard and dense endocarp (tegument), which contains the seed (almond) adhered to the endocarp [50].

The main seed predators of *A. aculeata* Bruchinae beetles belong to the tribe Pachymerini, originated in the Americas. These beetles use palms almost exclusively as a host plant and are usually host specific [38], [40], [51]. With respect to *A. aculeata*, individuals of *Pachymerus cardo* (Fahraeus 1839) and *Speciomerus revoili* (Pic 1902), also cited in literature as *Caryobruchus acrocomie*, have been identified as seed predators [37], [39]. *P. cardo* was recorded living alone or co-occurring with other species of Bruchinae in several palms [41]. The species *A. aculeata* and *P. cardo* used in field studies are not classified as endangered or protected.

Bruchinae females lay several eggs in a single night, mostly in fruits that have fallen on the soil [52]. The egg-laying occurs on the exposed portion of the fruit (epicarp, mesocarp, or endocarp), and an overlap of predator generations may occur. Approximately five days after egg-laying, the larvae emerge from the eggs and perforate the fruit towards the endosperm (author’s personal observation). While several larvae penetrate the endocarp only one individual per seed survives until adulthood [37], [40], [52]. The endosperm and the embryo are completely used as resources for the larval development. The insect emerges from the seed, only as an adult, through an exit orifice of approximately 6 mm made by own insect [37], [40], [41], [52]. Adult insects visit flowers during the day to feed on nectar and pollen [53].

### Prediction i: Injuries Caused by Herbivores in the Epicarp of Fruits during Pre-dispersal Favor the Colonization of the Fruit by Decomposing Fungi

#### Study site

The experiment was performed on the campus of the State University of Montes Claros (in Portuguese, Universidade Estadual de Montes Claros, Unimontes), in the municipality of Montes Claros, north of Minas Gerais State, southeastern Brazil. No specific permissions are required for experiments in this area. According to data from the Institute of Applied Geosciences (in Portuguese, Instituto de Geo-Ciências Aplicadas [IGA]), the head office of the municipality has an altitude of 638 m and is located at the coordinates 16° 43′ 41″ N, 43° 51′ 54″ W. Vegetation at the study site is composed by Cerrado (Brazilian savanna) and Caatinga (xeric scrubland). The climate is tropical semi-arid, hot and dry, with a period of concentrated rainfall between October and March, according to the Municipal Secretariat of Economic Development, Tourism, Science, and Technology [54]. The mean annual precipitation is 1,060 mm, and the mean annual temperature is 24.2°C [55].

#### Sampling design

To test prediction **i**, 200 intact fruits of *A. aculeata* at the final stage of the maturation process were collected from the clusters of three individuals located at Unimontes in December 2012 and were brought to the Laboratory of Ecology and Biological Control of Insects. The fruits were divided in to two groups, designated “treatment” and “control”. For the control group, 100 fruits with an intact epicarp with no sign of herbivory were selected. To simulate herbivory, which occurs during pre-dispersal, all fruits from the treatment group had the epicarp perforated at three points using a steel pin that was 0.5-cm thick and was sterilized by fire before each perforation, thus exposing the mesocarp.

At the end of December 2012 (rainy season), five individuals of *A. aculeata* in the reproductive stage were selected. Under the canopy of each selected tree, twenty fruits of treatment group and twenty fruits of control group were placed. Each group was covered by an exclusion cage measuring 0.20×0.60×0.60 m and with a 0.01-m metallic mesh. Our sampling design therefore comprises of five cages for the treatment group and five cages for the control group. Exclusion cages were used to prevent vertebrates of removing fruits used for the experiment, only allowing the access of insects. After 7, 14, 28, and 35 days of exposure in the field, 5 fruits from the treatment group and 5 fruits from the control group were collected of each select tree. The fruits were brought to the laboratory in order to identify (through visually observation in the epicarp and mesocarp) the presence of fungi in both groups, treatment and control. Fruits that were colonized by fungi were sent to the Laboratory of Environmental Microbiology for identification and cultivation of the fungi.

#### Data analysis

The data obtained in the experiment was analyzed with the aid of generalized linear models using the software R (version 2.15.3), followed by residual analysis to verify the adequacy of the error distribution and the model fitting [56]. The complete models were simplified, whenever necessary, with the removal of all non-significant variables and interactions (p>0.05) obtaining the minimum adequate model. The difference in the probability of fruit with fungal contamination among treatments was analyzed assuming a Binomial error distribution. In the model, presence or absence of contamination by fungi was considered the response variable, whereas the time of exposure (days) and the treatments were considered explanatory variables. The plant was considered as a block variable in the model in order to verify the effect of location on the results. Complete model included the interaction between the two variables analyzed.

### Prediction ii: Colonization of the Fruit by Decomposing Fungi Influences the Predator’s Selection of the Egg-laying Site

Two experimental approaches were used to test for the effect of fungi on the selection of the egg-laying site by species of seed predators of *A. aculeata*: **a)** field observations of the colonization by fungi and attack by insects on exposed fruits on the soil and **b)** experiments regarding the egg-laying preference and behavior of *P. cardo* females in the laboratory.

### Experiment (a): Colonization by Fungi and Attack on the Fruits of *A. aculetata*

#### Study site

Fields samples were performed in a private pasture area in the municipality of Mirabela, located in the north of Minas Gerais State, southeastern Brazil (16°15′46″S, 44°09′50″W). This region is characterized by a transition to a semiarid climate, with high temperatures and a pronounced dry season [57]. The municipality of Mirabela, is in the Cerrado (Brazilian Savannah) biome and has a mean annual temperature of 22.4°C, a mean annual precipitation of 1,082.3 mm, and a mean altitude of 862 m [55]. All land owners allowed access to their properties for completion of the experiments. This paper’s corresponding author should be contacted for future permissions.

#### Sampling design

For the field observations, 10 individuals in reproductive stage of *A. aculeata* were selected, with approximately 50 m between each pair of individuals. Four exclusion cages measuring 0.20×0.60×0.60 m, covered in a 0.01-m metallic mesh, were placed under the canopy of each individual at a height of 0.50 m on the trunk. Exclusion cages were used to prevent vertebrates from removing the fruits used for the experiment, only allowing the access of insects. Each cage contained 20 frutis of *A. aculeata* in its interior, for a total of 80 fruits per plant. The fruits used in this experiment originated from a sub-sample of 800 fruits haphazardly collected from the clusters of *A. aculeata* individuals from seven populations located in the state of Minas Gerais. To reduce the interference from the genetic and biometric characteristics of the fruits on the results, the fruits of different populations were mixed to obtain a composite sample.

The evaluation for the presence of fungi and eggs in fruits of *A. aculeata* ocurred from April to June 2012. Fruit collections were performed after 30, 45, 60 and 75 days of exposure, at each collection, five fruits were randomly removed from each cage, for a total of 200 fruits. In the field, the number of eggs per fruit and signs of fungal colonization were recorded. The fruits that had eggs and/or fungi were individually placed in plastic jars with lids and labeled. Fruits that contained eggs were incubated in a refrigerated BOD incubator (Cienlab model CE-300/350F) at 25.0°C, without photoperiod, to wait for the emergence of the adults in order to determine which species were found preying the fruit (*P. cardo* or *S. revoili*). Fruits that contained fungi were sent to the Laboratory of Environmental Microbiology for fungal identification and cultivation.

#### Data analysis

Data analysis was performed using generalized linear models followed by the analysis of residuals using the software R (version 2.15.3). A model was created to test for the effect of fungal presence on the egg-laying by seeds’ predators. In this model, the number of eggs found per fruit for each plant was considered the response variable, whereas the period of exposure (days) and the presence or absence of fungi were considered explanatory variables. This model was analyzed assuming a poisson error distribution. Another analysis was conducted considering the presence or absense of eggs on fruits as probability of attack by seeds predator. For this, a model was created considering the presence or absence of eggs in the fruits as response variable and the period of exposure (days) and the presence of fungus as explanatory variables assuming a binomial error distribution. In both models (for number and presence of eggs) a quadratic equation was fitted as a parabolic tendency was observed for the relationship. The plant was considered as a block variable in the two models built in order to verify the effect of location on the results.

### Experiment (b): Egg-laying Preference

#### Sampling design

To test for direct effect of the olfactory signs potentially emitted by the fungus contaminated fruits on *P. cardo* females, an adaptation of the triple-choice arena of Karimzadeh *et al.* [18] was built. This arena was composed of a series of transparent plastic jars that were 10-cm in diameter and 16-cm deep. One jar was placed in the central position and was connected to three other jars of equal size by transparent plastic tubes that were 13-mm in diameter and 4.5-cm in length, arranged in an angle of 120° among the jars. The jars were covered with a 2-mm mesh, and the central chamber had a battery-charged cooler. The cooler was placed to force the air to circulate in an ascending movement, ensuring the homogeneous flow of the odors through the central chamber.

In each trial, one insect at a time was placed in the central chamber. The insect had equal access to the three choices: healthy *A. aculeate* fruits, *A. aculeata* fruits contaminated by fungi, and an empty jar (control). The experiment was performed in February 2013 always at night, when there is a higher insect activity, with the aid of a laminar airflow chamber and with the lights turned off. Each trial was considered a replicate, and the experiment consisted of 40 repetitions. Observations periods lasted for a maximum of three hours per replicate, occurring every 30 minutes, until the insect have made a choice. Insects that remained in the central chamber, not responding to the test within the maximum observation period were not considered for statistical analysis. For each new trial, the arena was disinfected with alcohol at 70% v/v, followed by three rinses with water, to eliminate odors and possible fungal spores.

The individuals of *P. cardo* used in this experiment were obtained from the breeding Bruchinae kept in the Laboratory of Ecology and Biological Control of Insects at Unimontes and the females during their mating period were selected. The fruits used in the experiment were all collected under the crown *A. aculeate* individuals in the campus of Unimontes. All fruits collected were intact with no marks of herbivory, and had recently fallen from the mother plant. The fruits that composed the sample “healthy fruits” were disinfected with a cloth soaked with hypochlorite solution at 0.8% v/v to eliminate possible spores of fungi and were kept in plastic bags until the time of the experiment. The fruits that composed the sample “fruits colonized by fungi” were kept in plastic bags and left in a hot, humid, and dark place for one month to make the fungal colonization evident at the time of the experiment.

#### Data analysis

Data obtained from the experiment were analyzed for the choice and egg-laying behavior and were grouped according to the repetitions and compared with a null hypothesis of random choice using the Chi-squared test with Yates’ correction factor. The expected values were considered as a same proportion for each choice.

## Results

### Prediction i: Injuries Caused by Herbivores in the Epicarp of Fruits before Dispersal Favor Colonization of the Fruit by Decomposing Fungi after Dispersal

**Figure 1:**
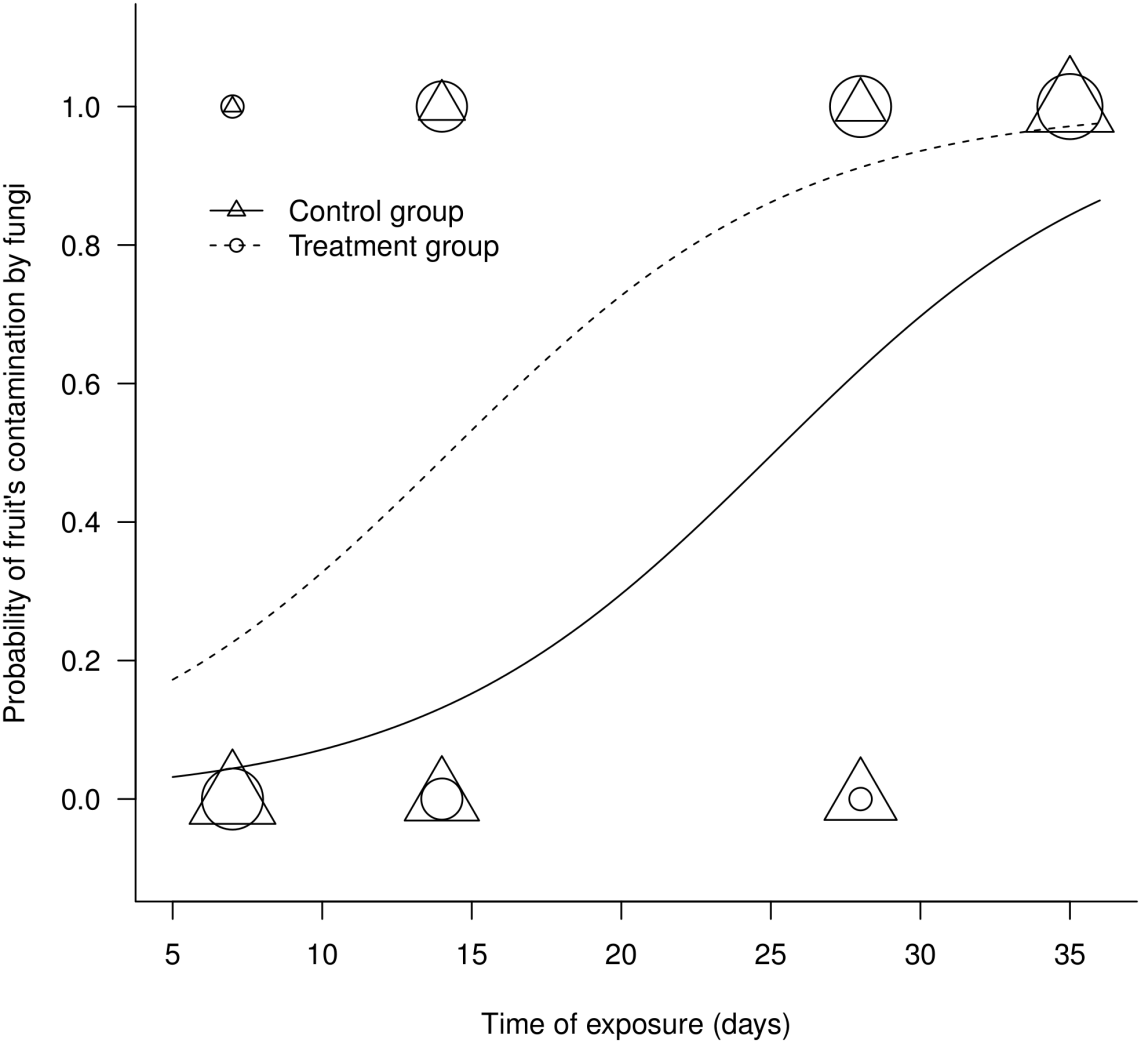
Effect of the exposition of mesocarp (treatment) and of the period of exposure on the probability of fruits colonization by fungi. The size of the symbols represents the number of overlapping points.

At the end of the experiment, out of the 200 fruits used, 53% were colonized by fungi. A total of 102 morphospecies of fungi were identified from the fungi that were isolated from the fruit epicarps and mesocarps. These morphospecies are from the genera *Aspergillus, Fusarium, Rhizopus, Penicillium, Cladosporium, Acremonium, Memnoniella, Spermospora,* and *Scedosporium.* The genera *Fusarium* and *Aspergillus* had the highest abundance. The treatment group, which consisted of fruits that were perforated to simulate herbivory in pre-dispersal, had a higher rate of fungal colonization than did the control group, which consisted of intact fruits. Colonization of the fruits by decomposing fungi was significantly influenced by the time of exposure of the fruits in the field (deviance_[1,198]_ = 89.719; p<0.001) and by the treatment type (deviance_[1,197]_ = 20.669; p<0.001) (Fig. 1). This result corroborates the proposed prediction. The interaction of these variables and the plant block variable does not significantly explain the presence of fungus in the fruits (p = n.s.). Although the treatment group had a higher rate of fungal colonization, both the treatment and the control group displayed an increasing tendency in the colonization rate until approximately 20 days of exposure. After this period, the rates tended to stabilize. All the fruits that remained exposed until the last collection (after 35 days) were colonized by fungi (Fig. 1).

### Prediction ii: Colonization of the Fruit by Decomposing Fungi Influences the Predator’s Selection of the Egg-laying Site

#### Experiment (a)

**Figure 2:**
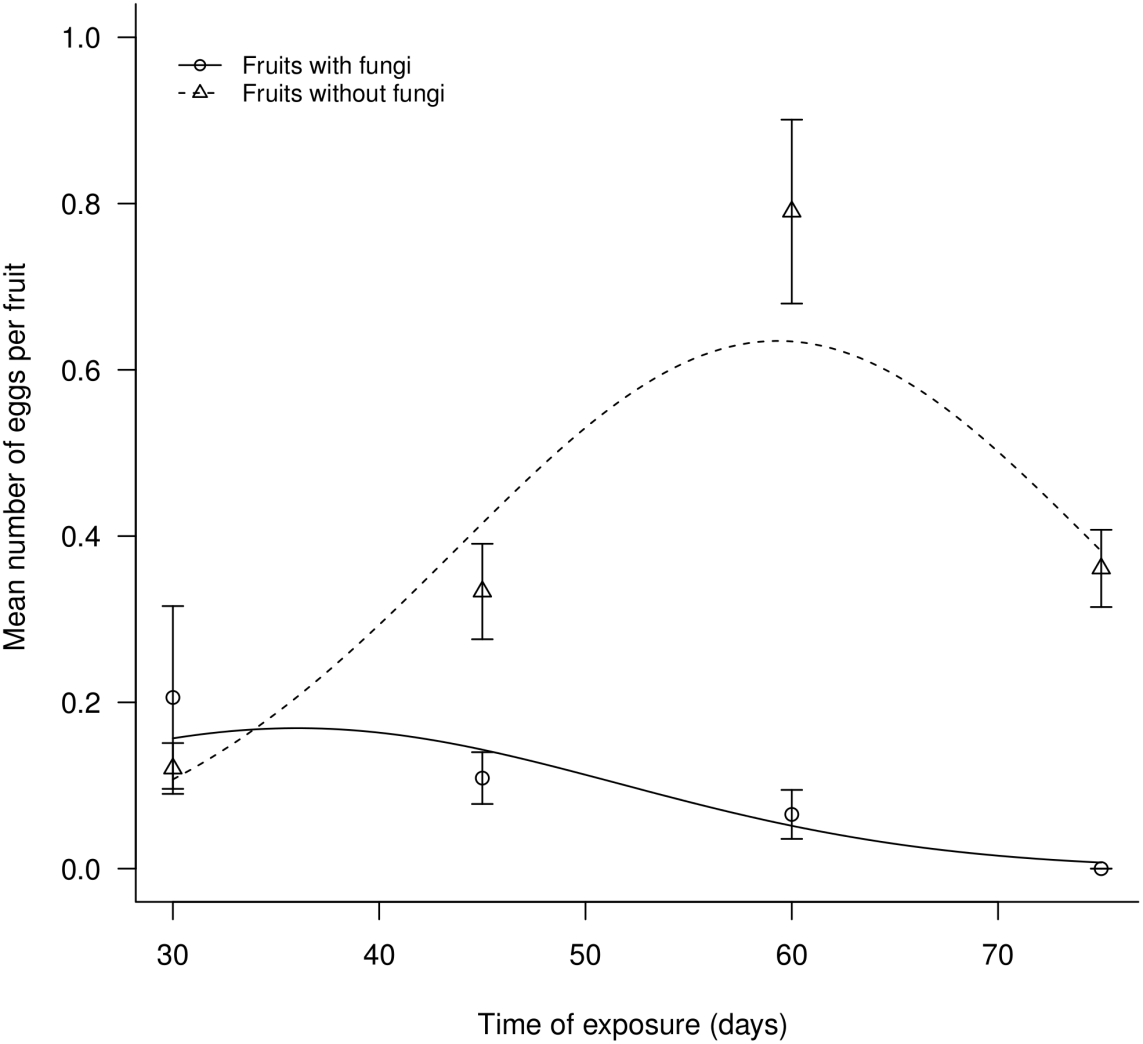
Effect of the presence of decomposing fungi on the number of eggs of Bruchinae in *A. aculeata* fruits by over time.

Of the 800 fruits used in the experiment, five were excluded, as in the last collection the exclusion cage was accidentally removed, allowing the access of cattle to fruit. Of the remaining 795, 19% contained eggs of seeds predators – in the egg stage it is impossible to determine species (only the subfamily – Bruchinae – can be assured). The maximum and minimum number of eggs per fruit was 3 and 0, respectively, with a mean of 0.24 eggs per fruit. Fruits in which fungi were not observed had a mean number of eggs that was approximately 3 times higher than that of fruits colonized by fungi. The mean number of eggs per fruit was significantly influenced by the time of exposure of the fruits in the field (deviance_[1,793]_ = 18.135; p<0.001) and by the presence of fungi (deviance_[1,792]_ = 44.924; p<0.001) (Fig. 2). The time of exposure takes a quadratic behavior about to mean number of eggs (deviance_[1,791]_ = 22.534; p<0.001). The interaction of the variables presence of fungi and time of exposure significantly explained (deviance_[1,790]_ = 22.541; p<0.001) the difference in the mean number of eggs. The presence of fungi reduced the mean number of eggs over time, which was evident throughout the entire exposure period (Fig. 2). By contrast, fruits without fungi had an increasing mean number of eggs until 60 days of exposure (Fig. 2). After this period, there was a decrease in the rate of egg-laying by Bruchinae in fruits without fungi. The block variable plant was non-significant for results found (p = n.s.).

**Figure 3:**
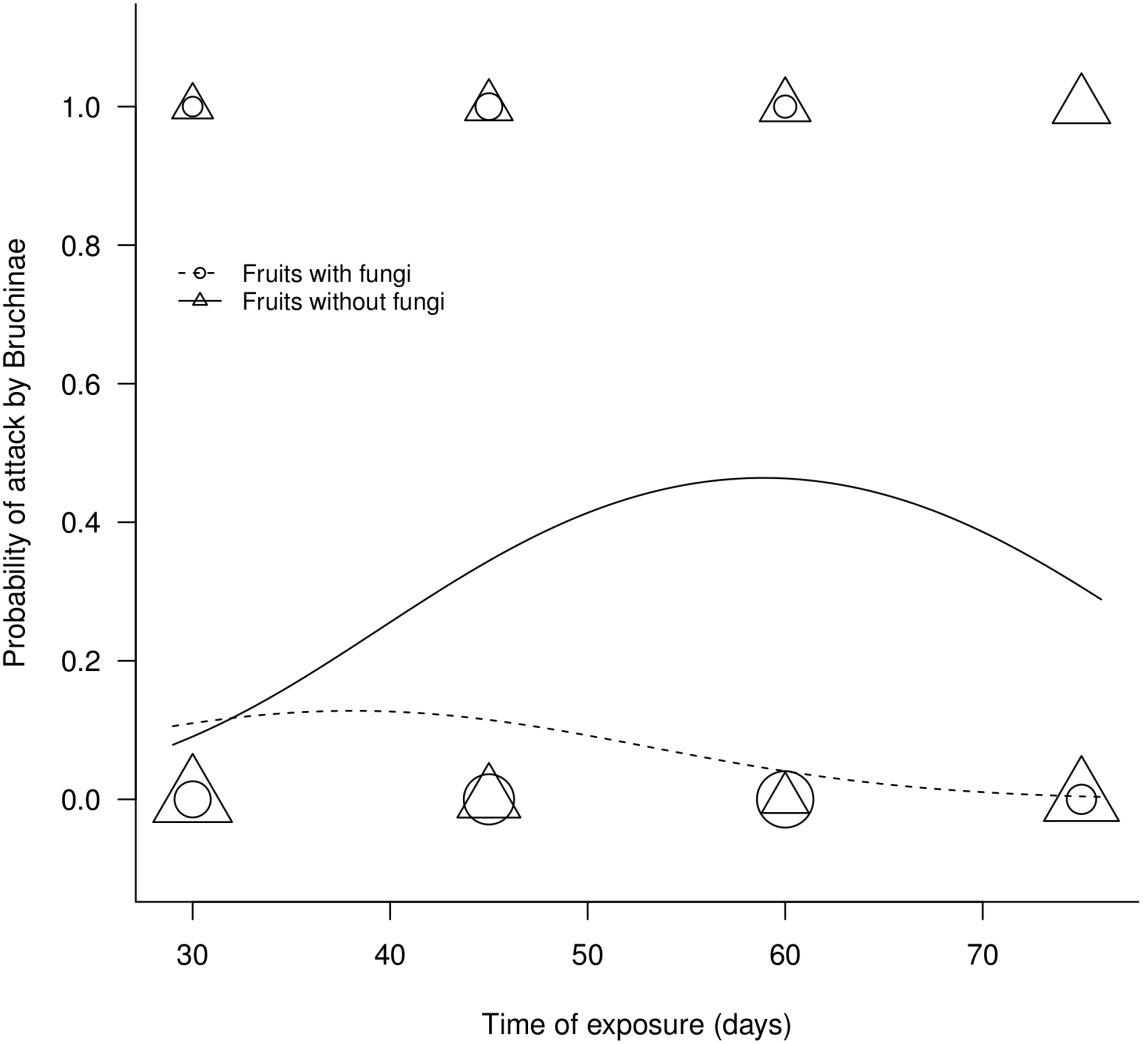
Probability of attack by seeds predator in *A. aculeata* fruits with or without fungi over time. The size of the symbols represents the number of overlapping points.

The attack (probability of finding eggs of the seeds predators in the fruits) varied from 0 to 56% per plant in the collections. This was significantly influenced by the time of exposure of the fruits in the field (deviance_[1,793]_ = 16.663; p<0.001) and by the presence of fungi (deviance_[1,792]_ = 45.286; p<0.001), being more frequent in fruits that did not have fungi (Fig. 3). The time of exposure assumes a quadratic behavior about to attack by seed predators (deviance_[1,791]_ = 21.138; p<0.001). The interaction between the variables time of exposure of fruits and presence of fungi also was significant (deviance_[1,781]_ = 20.245; p<0.001). The variable block plant was significant (deviance_[9,782]_ = 36.683; p<0.001), however, not changing the results found for the influence of others variables on the probability of attacked fruits.

#### Experiment (b)

**Figure 4:**
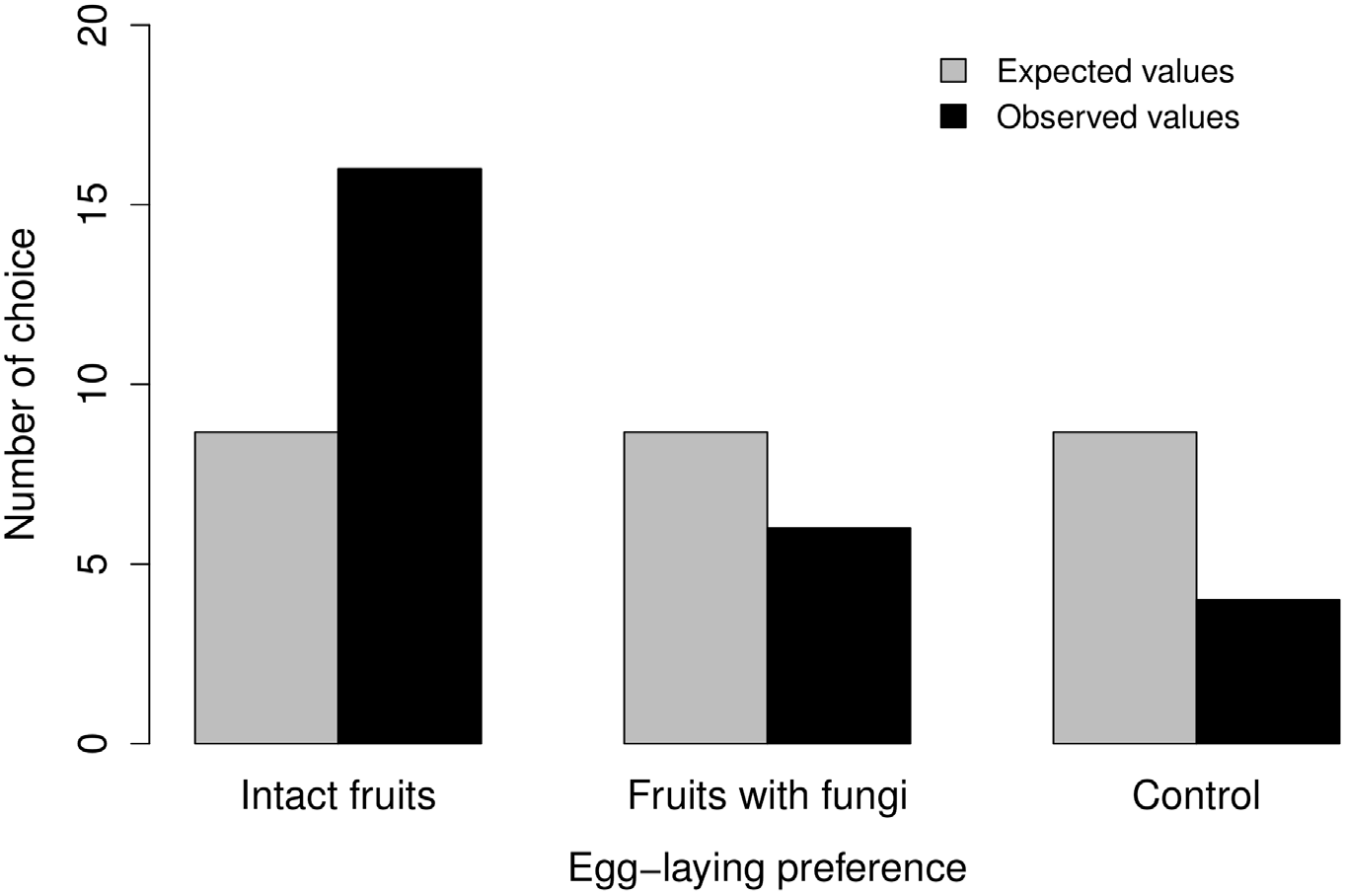
Results for each choice in the triple-choice arena: expected and observed values.

In the experiment regarding egg-laying preference and behavior, the beetle did not respond in 15 of the 40 repetitions, remaining in the central chamber until the maximum observation time was reached. Of the insects that responded to the experiment, approximately 54% of the choices occurred within the first 30 minutes of observation. The mean response time was significantly different among the choices (p<0.001, deviance_[1,23]_ = 86.085). The mean time of choice was 67 minutes for healthy fruits, 70 minutes for fruits with fungi, and 112 minutes for the control jar. This result indicates that the insects that opted for the empty jar were not influenced by odor in their choice. *P. cardo* females showed a preference for healthy fruits (x^2^_[2]_ = 9.5385, p<0.01) (Fig. 4). Of the 26 repetitions for which there was a response by the insect, the frequency of choice was 65% for intact fruits, 23% for fruits colonized by fungi, and 15% for the control jar.

## Discussion

Fungi are abundant in the soil and exert an important influence on the dynamics of seeds on the soil [58]. Once the fungi in this study were isolated from fruits of *A. aculeata* exposed while on the soil, most of the genera found correspond to soil fungi. *Aspergillus, Rhizopus, Penicillium, Cladosporium,* and *Fusarium* are commonly isolated from the soils of forests and fields, sandy areas, or cultivated areas [59], [60].

Fungi may directly affect seeds by facilitating germination [61], altering the survival of seedlings or causing the death of the embryos [62], [63]. Fungi can also indirectly affect the mortality of seeds by producing toxins that may reduce the subsequent microbial colonization and alter the pattern of seed attack by animals [63]–[65]. The direct and indirect interactions between fungi and seed survival are well known [66]–[69] and usually involve endophytic and pathogenic fungi. Indirect interactions with soil fungi are difficult to study because of the high diversity of this group of fungi, which includes mycorrhizae, saprophytes, mycoparasites, root pathogens, and other trophic types [68].

The rates of fungal colonization in fruits are influenced by characteristics such as the soil moisture [66], [70], host species [61], [67], soil type [71], [72], fungal density in the soil [73], and maturation stage of the fruits [65]. The present study showed that injuries caused by herbivores to the fruits of *A. aculeata* are also capable of influencing fungal colonization. When the wall of the fruit is broken by abrasion, falls, damage by claws or teeth, partial consumption, or active entrance by an herbivorous insect, microorganisms begin a race to colonize the most nutritive part of the fruit [65], [74]. This pattern explains why the fruits with injuries, similar to those caused by herbivores during pre-dispersal, were more rapidly colonized than were intact fruits in this study (Fig. 1). However, the natural deterioration of fruits over time compensated for the exposure of the mesocarp by herbivory, resulting in similar colonization percentages for intact fruits and for fruits with injuries after 35 days of exposure (Fig. 1).

The level of infestation of the fruit may affect the specific responses of seed predators to the microflora and can be sufficient to cause a loss of seed viability [58], [64], [75]. For *A. aculeata*, the loss of seeds viability by fungi is limited by the difficultly in penetrating the fruit because of the thick and woody endocarp, which protects the seed and thus to the embryo (Author’s personal observation).

Herbivory of *A. aculeata* fruits during pre-dispersal negatively influenced the fruit predation during post-dispersal. Although herbivores and predators act on the same resource, they do not interact directly because they act during different stages of maturation and in different parts of the fruit. This behavior excludes a direct antagonistic relationship and renders possible the existence of an indirect interaction between the insects that attack *A. aculeata* fruits during pre- and post-dispersal. The present study suggests that soil fungi act as mediators in this interaction.

The presence of fungi on fruits affects the egg-laying behavior of the seed predator. The maximum number of three eggs per fruit is low considering that studies performed in Central Brazil have reported more than 12 eggs of Bruchinae per *A. aculeata* fruit [37], [39] and considering that a Bruchinae female may lay between 50 and 100 eggs per fruit [53]. Despite the low number of eggs per fruit, the present results indicated a decrease in the mean number of eggs with an increase in fungal colonization of the fruits. In the choice experiment, the *P. cardo* females showed a clear preference for intact fruits after mating (Fig. 4). An influence of fungi on egg-laying was found for the beetle *Cassida rubiginosa* Müller (Chrysomelidae: Cassidinae). The feeding and egg-laying behaviors of the adults in this species were negatively altered by the presence of fungi on the leaves of its host plant [76]. Aphids of the species *Rhodobium porosum* Sanderson (Hemiptera: Aphididae) also showed a reduction in population growth with the infestation of their host plant by pathogenic fungi [21].

The present results can be explained by the fact that Bruchinae females, before laying eggs, examine the surface of the fruit with their ovipositor. This organ possesses tactile receptors and chemoreceptors, which receive information on the fruit surface, such as the moisture and chemical content [77]. This information is used for choosing or refusing the fruit for egg-laying. Therefore, the presence of fungi in *A. aculeata* fruits protects the propagule from predation by Bruchinae, thereby reducing the availability of fruits for the predator. Several fungi are well known to produce toxins that can subsequently reduce the colonization of the seed by other microorganisms and the consumption of the seed by animals [58], [62]. Therefore, associations between plants and fungi can alter the direction of predator-prey interactions [23].

Despite the involvement of physical factors, such as the type, texture, size, and color of fruits, the selection of the egg-laying site depends primarily on semiochemicals that influence the efficiency of foraging [18], [78]. The action of the fungus on the fruit appears to generate a chemical alteration that hinders discovery of the fruit by a predator. In this case, during foraging and in the context of the diverse chemical signals found in the natural environment, the beetle cannot identify fruits with this altered pattern of semiochemicals caused by the presence of fungi. Alternatively, fungi might have a toxic effect on Bruchinae eggs, similar to that reported for fungi of the genus *Phomosis* on eggs of Scolitidae beetles [79]. In this case, the fungi exerted a two-fold effect on the beetles by decreasing the number of viable eggs per litter and by reducing the number of potential reproduction sites.

**Figure 5:**
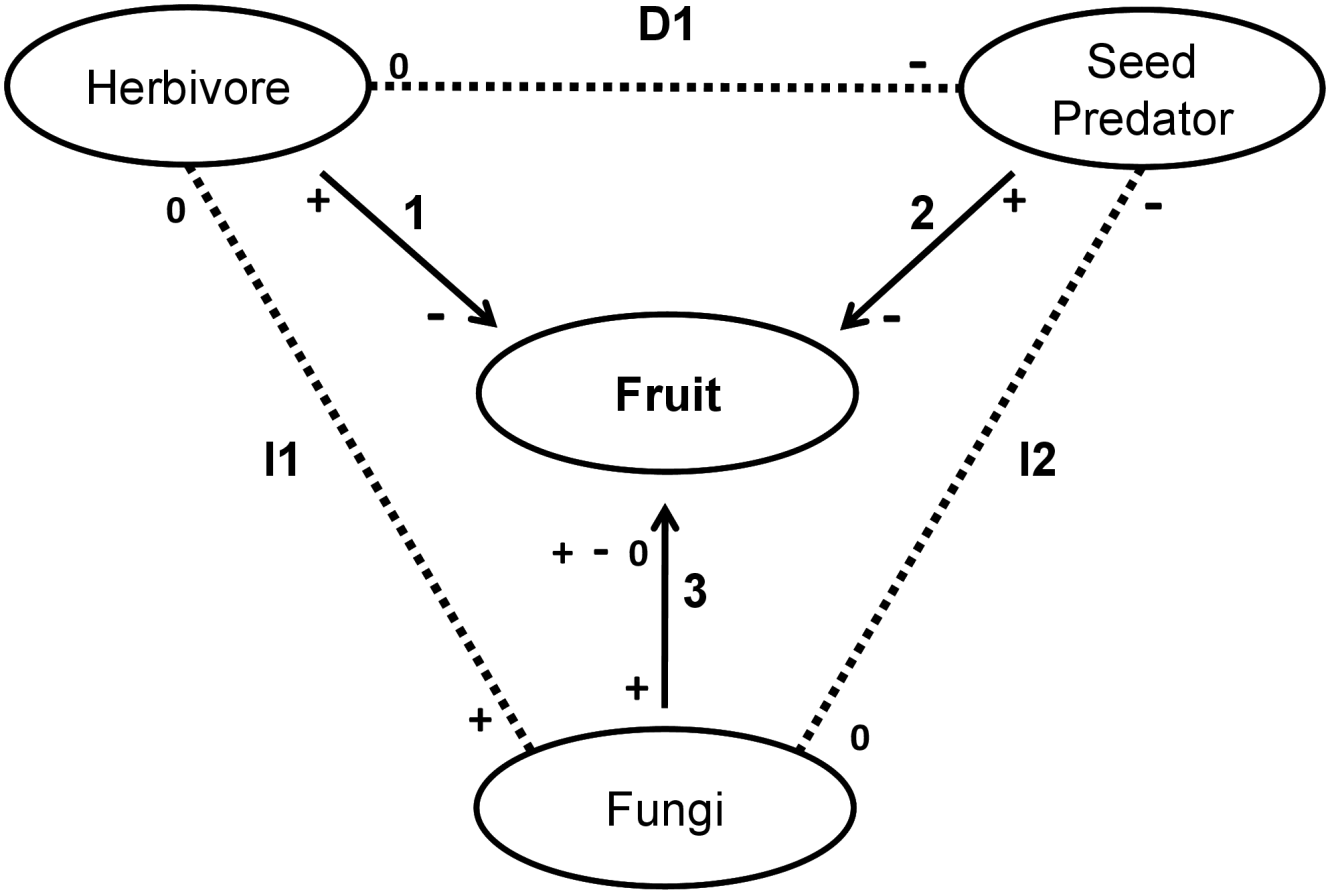
Network of interactions that involve *A. aculeata*. **1-** Direct interaction of fruit herbivory. **2-** Direct interaction of seed predation. **3-** Direct interaction of fruit decomposition. **I1-** Indirect interaction among the herbivore, fruit, and fungus. **I2-** Indirect interaction among the fungus, fruit, and predator. **D1-** Diffuse indirect interaction between the herbivore and the predator resulting from the indirect interactions (I1+I2).

Decomposing fungi mediated the interaction between herbivores and predators of *A. aculeata.* The fungi were opportunists with respect to the colonization of fruits that had their mesocarp exposed by the action of herbivores before dispersal. The presence of the fungus altered the egg-laying behavior of the main seed predator *A. aculeata*, benefiting the recruitment of the plant. Therefore, the dynamics of *A. aculeata* populations are subject to a complex network of direct and indirect interactions among several trophic levels (Fig. 5). These interactions include (1) direct action of the herbivore on the fruit during pre-dispersal, (2) attack of seed predators on the fruit during post-dispersal, and (3) colonization of the fruits by decomposing fungi which may be positive, negative, or neutral for the fruits, depending on the intensity of the colonization. Indirect relationships also arise in this system. The attack of the herbivore indirectly alters the rate of fungal colonization (I1), and the fungal colonization of the fruits alters the egg-laying behavior of the predator (I2). Therefore the interaction between herbivores before dispersal and predators after dispersal (D1) results from the two indirect interactions (I1+I2) and, in this study, is designated as a diffuse indirect interaction.

Diffuse indirect interactions, which are rarely addressed in studies of multitrophic interactions, can have a fundamental role in the structuring of natural populations. The direct action of an herbivore on a fruit can have little significance on the fitness of the plant. However, the diffuse relationship between the herbivore and the predator, mediated by the community of decomposers, has important repercussions on the fruit’s escape from predation and thus on the survival of the propagule. Therefore, the study of diffuse interactions is necessary for a complete understanding of the population dynamics of species in natural environments.
